# Selective Attention Shapes Neural Representations of Complex Auditory Scenes: The Roles of Object Identity and Scene Composition

**DOI:** 10.1523/JNEUROSCI.0506-25.2025

**Published:** 2025-10-14

**Authors:** Patrik Wikman, Ilkka Muukkonen, Jaakko Kauramäki, Ville Laaksonen, Onnipekka Varis, Christopher Petkov, Josef Rauschecker

**Affiliations:** ^1^Department of Psychology, University of Helsinki, Helsinki 00014, Finland; ^2^Department of Neuroscience, Georgetown University, Washington, DC 20057; ^3^Advanced Magnetic Imaging Centre, Aalto NeuroImaging, Aalto University, Espoo 02150, Finland; ^4^Centre of Excellence in Music, Mind, Body and Brain, University of Helsinki, Helsinki 00014, Finland; ^5^Cognitive Brain Research Unit, Department of Psychology, Faculty of Medicine, University of Helsinki, Helsinki 00014, Finland; ^6^Better Taste Music, Helsinki 00510, Finland; ^7^Department of Neurosurgery, University of Iowa, Iowa City, Iowa 52242; ^8^Institute of Neuroscience, Newcastle University, Newcastle NE17RU, United Kingdom

**Keywords:** auditory objects, auditory scene analysis, functional magnetic resonance imaging, multivariate pattern analysis, selective attention, top-down modulation

## Abstract

Everyday auditory scenes contain overlapping sound objects, requiring attention to isolate relevant objects from irrelevant background objects. This study examined how selective attention shapes neural representations of complex sound scenes in the auditory cortex (AC). Using functional magnetic resonance imaging, we recorded brain activity from participants (12 males, 8 females) as they attended to a designated object in scenes comprising three overlapping sounds. Scenes were constructed in two manners: one where each object belonged to a different category (speech, animal, instrument) and another where all objects were from the same category. Attending to speech enhanced activations in lateral AC subfields, while attention to animal and instrument sounds preferentially modulated medial AC subfields, supporting models where attention modulates feature-selective neural gain in AC. Remarkably, however, spatial pattern analysis revealed that the attended object dominated the AC activation patterns of the entire scene in a manner depending on both object type and scene composition: When scene objects belonged to different categories, attention effects were dominated by category-level processing. In contrast, when all scene objects shared the same category, dominance shifted to exemplar-level processing in fields processing acoustic features. Thus, attention seems to dynamically prioritize the features offering maximal contrast within a given context, emphasizing object-specific patterns in feature-similar scenes and category-level patterns in feature-diverse scenes. Our results support models where top-down signals not only modulate gain but also affect scene decomposition and analysis—influencing stream segregation and gating of higher-level processing in a contextual manner, adapting to specific auditory environments.

## Significance Statement

Selective attention is essential for filtering behaviorally relevant sounds from complex auditory environments, yet the underlying neural mechanisms remain obscure. We combined fMRI with spatial activation pattern analysis to determine how the auditory cortex attentionally filters different types of sounds (speech, animal, instrument) in complex scenes composed of three sounds, either from different or the same categories. Attentional filtering depended both on the object type and on scene composition. Our data suggest that in the auditory cortex attentional filtering operates on category-level features in multi-category scenes, while exemplar-level features prevail in same-category scenes. Thus, top-down attention not only modulates neural gain but also affects scene decomposition and gating of higher-level processing in a contextual manner, adapting to specific auditory environments.

## Introduction

Auditory objects—sounds assigned to specific sources like speech or birds—form auditory scenes (Kubovy and Van Valkenburg, 2001; Winkler et al., 2009). The representations of such objects become increasingly abstract in the hierarchical auditory ventral stream, proceeding from selectivity for acoustic features (e.g., frequency) in primary auditory cortex (AC), to acoustical structures in secondary AC, culminating in object representations in the anteroventral AC, and categorical representations in middle temporal gyrus (Rauschecker et al., 1995; Rauschecker and Tian, 2000; Boemio et al., 2005; Petkov et al., 2008; Rauschecker and Scott, 2009; Lewis et al., 2011; Theunissen and Elie, 2014). There is still, however, debate whether representations are instead distributed within AC subfields selective for different acoustic features (Formisano et al., 2008; Staeren et al., 2009; Giordano et al., 2023).

Cognitive functions such as attention and memory dynamically shape the processing of sounds in AC (Ahveninen et al., 2011; Backer and Alain, 2014; Michelmann et al., 2016). A key question is how attention affects auditory scene perception and neural representation (Bizley and Cohen, 2013). Although object formation processes and scene parsing occur pre-attentively (Sussman et al., 1999; Winkler et al., 2009), attention appears to enhance and modify these processes (Zatorre et al., 1999; Cusack et al., 2004; Cusack, 2005; Shamma et al., 2010). Especially, in natural scenes with overlapping competing auditory objects, attention is thought to play a pivotal role in forming stable object percepts (Shinn-Cunningham et al., 2017). Recent “cocktail-party” studies show that AC activity more accurately represents attended speech than ignored speech (Mesgarani and Chang, 2012; Wikman et al., 2024). However, it remains unclear if such effects generalize to other object categories (Hausfeld et al., 2018). Moreover, attention is known to modulate processing at the feature level (Fritz et al., 2003; Da Costa et al., 2013), object level (Alain and Woods, 1994; Backer and Alain, 2014), or both depending on the context (Shamma et al., 2010, 2011; Wikman et al., 2020; for a review, see Noyce et al., 2023). Yet, it is not fully understood how variations in natural-scene composition shifts attention toward feature- versus object-level processing.

**Figure 1.**
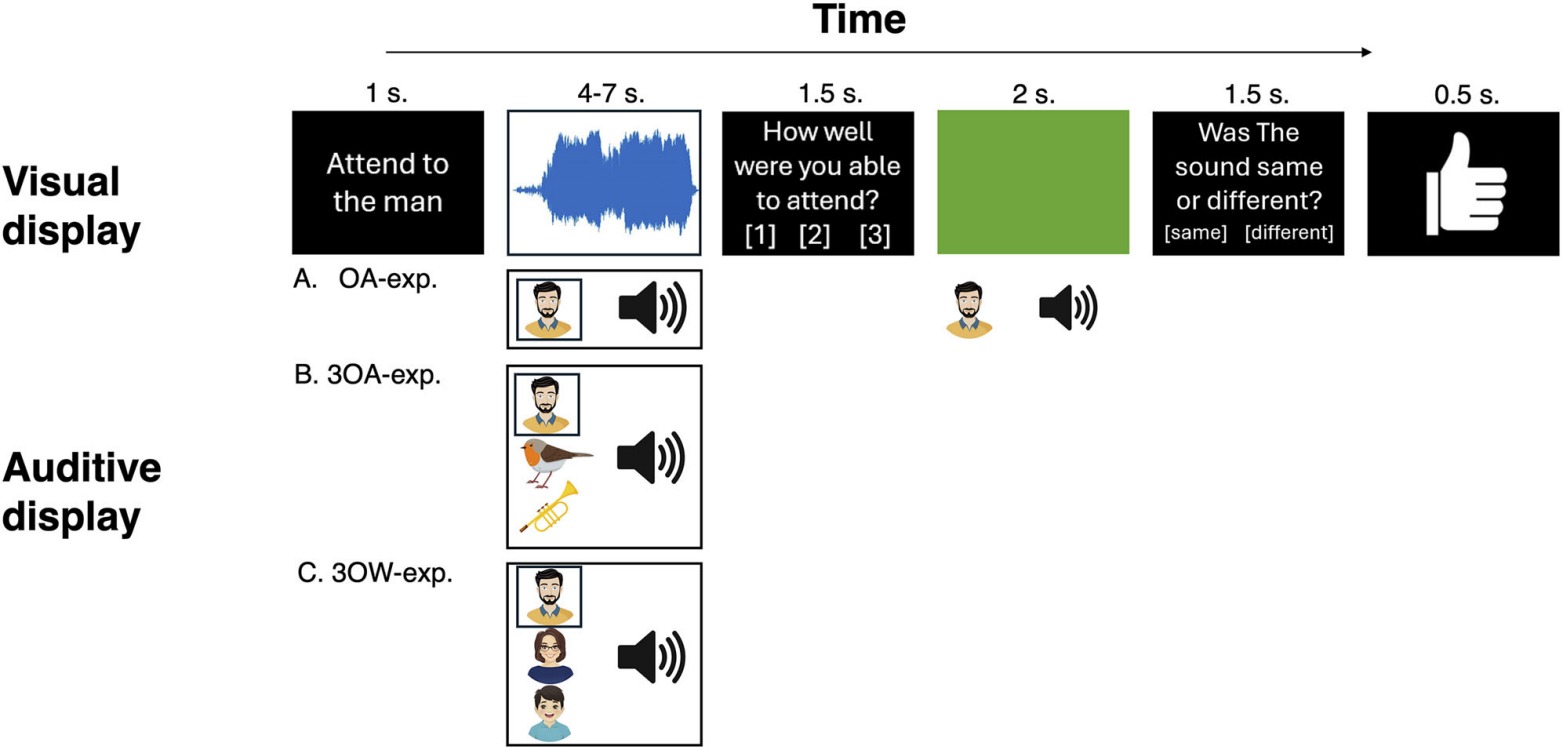
Behavioral paradigm used in the three experiments (OA, 3OA, 3OW). The trial structure was the same for all experiments, tasks, and stimulus types. The trial started with an instruction (1 s), orienting the participant to attend to a specific object. The attended object was either auditive (attend sound object task) or visual (visual control task). Thereafter the participant was presented with either one auditory object (OA), or three auditory objects (3AO or 3OW), as well as a visual video of a scrolling waveform display. Participants attended either to the designated sound object (male speaker in the schematic illustration, “attend to man”), while ignoring the video display, or to the video display while ignoring the auditory stimulation. After each trial, the participants rated their ability to attend to the designated stimuli and ignore the task irrelevant stimuli (1, <33% of the time; 2, 33–66% of the time; and 3, >66% of the time). After some trials the participant also performed a match-to-sample task, where a short sample of either the attended stimulus or another sample was presented. The participant was then tasked to report whether the sample was sampled from the previous attended stimuli or not. Lastly the participant received feedback on their performance.

Using fMRI, we studied whether selective attention to distinct natural sound classes differentially modulates AC processing. We presented natural sound clips from three sound categories (speech, animal, instrument) to 20 participants in three experiments (Fig. 1). Participants focused on one of three overlapping auditory objects. The overlapping objects were drawn from different categories (three objects across, 3OA experiment) or the same category (three objects within, 3OW experiment). The experiments were designed to answer key questions on the role of selective attention in auditory scene analysis: (1) At which hierarchical level does attention operate in complex scenes with overlapping natural sound objects? We hypothetized that when objects share acoustic features (3OW), attention would emphasize exemplar-level processing, but when objects are acoustically distinct (3OA), it would emphasize categorical level processing [see Cusack et al. (2004), hierarchical decomposition model for a related notion]. To test this, we compared attention-related modulations (ARMs) between 3OW and 3OA. Additionally, we used voxel-pattern regression [see Hausfeld et al. (2018, 2024) and Puschmann et al. (2024) for similar approaches] to evaluate whether the attended-object pattern dominates that of the entire scene and whether the hierarchical level of such modulation depends on scene type. To isolate each object’s activation pattern we, therefore, conducted a third experiment (object-alone, OA) where each sound object was presented in isolation. (2) We further asked whether attention simply boosts gain of neurons tuned to the attended object stimulus properties (Woldorff et al., 1993; Hillyard et al., 1998; Zion-Golumbic et al., 2013) or instead sharpens selectivity for whole-object representations (Petkov et al., 2004; Rinne et al., 2011). Thus, we tested whether attending to a sound category modulates the same AC fields that processes its stimulus properties and whether such ARMs can be explained with low-level acoustic features.

## Materials and Methods

### Participants

Twenty healthy right-handed monolingually native Finnish-speaking participants underwent an fMRI session (8 females, age range 19–32, mean age 22.7 years, standard deviation 3.68). All participants were adult university students at the University of Helsinki and Aalto University, self-reported normal hearing, and normal or corrected-to-normal vision and no history of developmental or neurological disorders. We assessed handedness by using the Edinburgh Handedness Inventory (Oldfield, 1971) and acquired written consent from the participants before the study. Sample size was based on our prior studies using uni- and multivariate fMRI analysis on data where participants selectively listened to overlapping speech (Wikman et al., 2020, 2022, 2024).

### Ethics statement

For their participation, the participants were compensated monetarily €15/h. Each participant provided written informed consent. Written informed consent was obtained for the sharing of processed anonymized data from each participant. The experiment was accepted by the University of Helsinki Ethical Review Board in the Humanities and Social and Behavioural Sciences, and we conducted the study in accordance with the Declaration of Helsinki.

### Data availability statement

All data (behavior and fMRI) preprocessed to anonymize and original code have been deposited at Open Science Framework under Attention and Memory networks (https://doi.org/10.17605/OSF.IO/AGXTH).

### Stimuli

The auditory stimuli comprised sound clips from three sound object categories: speech, animal, and instrument sounds. Each sound category included six subcategories. The speech subcategories comprised sound samples from two females, two males, and two children (one boy and one girl). The animal subcategories were sound samples from huskies, cats, whales, monkeys, birds, and seals. The instrument subcategories were sound samples from a baritone guitar, a 12-string guitar, a saxophone, a trumpet, a church organ, and a jazz organ. Eight sound object clips (exemplars), from each subcategory, were used, making the total amount of different auditory clips 144 [three categories (speech, animal, instrument), six sound objects for each category, and eight exemplars from each sound object]. The average duration of a sound sample was 5.3 s (4–7 s).

Speech samples were extracted from spoken dialogues recorded for our previous studies (Leminen et al., 2020; Ylinen et al., 2024), where details are given about the recordings. The dialogues were related to emotionally neutral everyday situations. The speech samples were segmented into 4–7 s audio clips with Audacity (version 3.3.3), each comprising one sentence.

Animal sounds were collected from free internet sources. The sounds were selected based on the versatile range of produced sounds: For example, a howling husky with changing pitch was preferred over a monotonously barking dog. Soundtracks with as little background noise as possible were adopted for further processing. Selected sounds were processed in Logic Pro X with Waves X-Noise broadband noise reduction plugin dampening the noise by 20–50 dB depending on the sample.

Instrument sound samples were created using clips from the same speakers as those used for the speech sounds, separate speakers being used for each specific instrument. Specifically, male voices were paired with 12-string guitar bright and baritone guitar samples, female voices with alto saxophone and trombone samples, and child voices with jazz organ and church organ samples. The selection of instruments corresponding to each speaker was determined by the frequency characteristics of their voice, ensuring the best match between voice and instrument. For instance, a baritone guitar was chosen to correspond with a low male voice, as its natural frequency range aligned well with that of the voice. Initially, the frequency and duration of each dialogue was analyzed using the Melodyne plugin within Logic Pro X. Subsequently, the frequencies were adjusted to conform to the 12-Tone Equal Temperament (12-TET) scale, and MIDI files were generated for each dialogue. To ensure coherence, outlier frequencies in the original dialogues were adjusted to align with the general frequency range of each dialogue. Following this, the audio files exported from MIDI files underwent compression to ensure consistent volume (velocity in Logic Pro X) and timbre across notes. Additionally, an Audacity compressor was applied to further compress the files, with parameters set to a threshold level of −30 dB, noise floor of –35 dB, ratio of 5:1, attack time of 1.11 s, and release time of 1.0 s. The instrument samples utilized were original samples from Logic Pro X and Spectrasonics Omnisphere except for organ samples sourced from the Logic Vintage B3 Organ plugin.

All auditory stimuli were of high quality (48 kHz sampling rate and 16 bit). If the sounds had excessive internal variability, they were compressed with the Audacity compressor. After compressing, silent parts from the beginning and end were removed, and a slight Fade-in and Fade-out (50–100 ms) was applied in Audacity. If there was noise in the audio clips, they were edited with the Auto-heal tool from Adobe Audition (version 22.6.0.66). The auditory stimuli were normalized using Matlab. Each audio file was scaled to adjust its average loudness to a target level measured in sones. The scaling was performed using the Matlab R2022b acousticLoudness() function, which returns loudness in sones according to ISO 532-1 (Zwicker) standard. The Matlab script determines the optimal scaling factor by minimizing the difference between the original and target loudness, computed as the average loudness across all audio files, using the fminsearch function. The audio signal was then scaled accordingly using the calculated factor. Only channel 1 was utilized in the computation. Finally, we filtered all sounds using the filter bank provided by Sensimetrics (model S14; Sensimetrics), to make the sounds optimal for the headphones used during fMRI (see below, Equipment and Stimulus delivery). At least 12 sound object exemplars were initially generated, of from which eight were used in the actual trials. Out of the remaining four samples, one was used for the practice and the other three for the match-to-sample task (see Pretrial).

The visual videos were created from all the auditory stimuli used in the experiment (214 sounds including the training sounds and sounds used as nontargets in match-to-target task). Using Matlab (R2020a, MathWorks), for each sound a scrolling waveform display was generated with 1 s window visible (blue waveform on white background, single audio channel normalized to maximize display size, concatenated twice and audio padded by a second of silence to have a smooth ending). Audio concatenation was done so that the generated videos would not end before the auditory stimulus presentation: Video duration always outlasted the auditory stimulus during simultaneous presentation. The single waveform display frames were cropped to 876 × 656 pixel dimensions. From these frames, 25 FPS silent videos were created in AVI container format using ffmpeg and MPEG4 video codec with 2,000 kbps target bit rate; AVI was used for compatibility issues. The resulting videos were manually screened to remove visually too similar videos, resulting in altogether 180 different control videos used in the experiment, presented independently of the auditory stimulus.

### Trial structure

Participants underwent three separate experiments (object-alone, OA; three objects across, 3OA; three objects within, 3OW). The main differences between the experiments were that OA included only one sound object per trial, while 3OA and 3OW had sound scenes comprising three overlapping sound objects, which were chosen across the three categories of sounds (speech, animal, instruments) in 3OA and within the three object categories in 3OW.

3OA was conducted to reveal how selective attention modulates processing when attending to objects in scenes with different categories of objects. 3OW was conducted to reveal how selective attention modulates processing in scenes with objects from the same object category. The OA experiment was used for behavioral performance (see Results, Behavioral performance) and to yield spatial activation patterns for single objects (see below, Spatial pattern analysis).

All three experiments (OA, 3OA, 3OW) used the same core trial structure, which can be divided into four parts: (1) An instruction was presented indicating whether the participant performed an auditory task (attend sound object task) and the identity of the sound object the participant was to attend to (e.g., “attend to the bird”) or whether the participant performed a visual task (visual control task, “attend to the visual display”). The instruction was displayed for 1 s. (2) After the instruction, the participant was presented with one sound object in OA (e.g., bird or male speaker) and three simultaneous sound objects in 3OA and 3OW. In addition, a randomly sampled (without replacement) visual video (4–7 s) was presented for the same duration as the auditory sounds. (3) Following the stimulus presentation, the participant was asked to give a subjective rating on how well they had been able to attend to the designated object by pressing a button (1.5 s response window) with an appropriate option: 1, <33% of the time; 2, 33–66% of the time; and 3, >66% of the time. (4) Lastly, in 25% of attend sound object task trials, the participant was asked to complete a match-to-sample task. In the attend sound object match-to-sample task, a short sample (2 s) of either the sound object exemplar they had been attending to was presented (target trial, 50% probability) or another exemplar of the same sound object (nontarget trial, clipped from one of the four sound object exemplars that were not used as attended stimuli) was presented (1.5 s response window). To alert the participant to the match-to-sample task, the background of the visual display was changed to green before (1 s) and during the presentation of the sample. After answering, the participant received feedback on their performance (0.5 s; correct/incorrect). The visual control match-to-sample task was otherwise identical to the auditory version, except instead of auditory clips, the participant was presented with a short sample (2 s) of either the video clip they had been attending to (target trial, 50% probability) or another video clip (nontarget trial; 1.5 s response window). To ensure participants did attend to the visual display in all visual control tasks the match-to-sample task was presented in 100% of trials instead of 25%. The intertrial interval was randomized to last from 201 to 5,500 ms.

### Experiments

In the OA experiment, the participants performed only one type of task: In the attend sound object task, participants attended to the presented auditory object, which was always presented alone without any other sound objects, whereafter they gave a subjective rating of how well they could focus on the sound and thereafter performed a match-to-sample task (see above, Trial structure, for details). Note that to make the stimulus presentation comparable across the three experiments, visual videos were also present in the OA experiment, although participants were never instructed to pay attention to them in the experiment. For each participant, each sound object exemplar (altogether 144) were presented in a separate trial. However, the presentation was split into four consecutive runs (36 exemplars/run) and each run always comprised two trials of each sound object type. The order of trials was randomly generated for each participant. For each sound object type there was one match-to-sample task where the sample was the same as the attended target sound object and one where it was not. As the match-to-sample task was presented for 25% of the exemplars in one run, there were nine auditory match-to-sample tasks in each run.

In the 3OA experiment, unlike the OA experiment, a sound object scene consisting of three sound objects, each from different sound categories (speech, animal, instrument), were presented to the participant (e.g., boy, husky, and trumpet). The participant performed two tasks (attend sound object task or visual control task). In this experiment, during the attend sound object task, the nonattended sound objects acted as distractors for the designated sound object which was to be attended. Accordingly, the participants were instructed prior to the experiment to not only attend to the designated sound object but to also actively block out all distractors (auditory and visual). To help the participant with orienting to the designated sound object, its presentation started 250–500 ms prior to the distractor sound objects. In the visual control task, participants were instructed to fully ignore all auditory stimulation and attend to the visual video. The attend sound object task was performed 75% of the trials and the rest were visual control task trials.

In the 3OA experiment, sound objects for each trial were combined accordingly: All sounds were selected between sound object categories (speech, animal, instrument) and randomized individually for every participant. Every sound object type (e.g., Male1) was presented in two different auditory scenes during the attend sound object task (e.g., see A for each subcategory in Table S1). Every sound type acted twice as a target, once in both auditory scenes. Even though the same sound objects were presented together multiple times, the exemplar was always a different one so that in each run 108 exemplars were used only once during the attend sound object task (12 different types of scenes × 3 sound objects per scene × 3 different attended object conditions). The combinations were generated by arranging sound objects into three rows stacked vertically and then rotating the row of instruments one step and the row of animals two steps (for demonstration see bolded parts in Table S1). Because there were three possible task allocations of each sound object (attended, distractor 1, distractor 2) and two possible scenes for each of the six subcategories, the number of trials of attend sound object task were 36 in each run (3 attended objects × 12 sound scene types). To have a control for each sound object scene, we additionally generated visual control task sound object scenes, each with the same sound objects as in the attend sound object task but different exemplars for each scene. Thus, we used the remaining 36 sound object exemplars for the visual control task (12 different types of scenes × 3 sound objects per scene). The exact same auditory scenes were presented in every run, but their order was randomized for every run.

The trial structure and tasks for the 3OW experiment were the same as those for *3OA*. However, in this experiment three sound objects were always from the same category of sound objects (e.g., Male1, Boy, Female2 or Bird, Husky, Whale). As in the 3OA experiment, each sound object type was presented in two different auditory scenes. However, in the 3OW experiment, the allocation of sound objects to their auditory scenes could not be generated in the same way as in the 3OA experiment. Thus, the combinations were generated within each sound category (e.g., animal) first by randomly shuffling the order of the sound object types. Thereafter the sound objects were chosen to a scene, in a manner so that each sound object was presented in two and only two auditory scenes. This resulted in four possible subsets for each participant (Table S2). Thus, there were three possible task allocations of each sound object (attended, distractor 1, distractor 2) and four different sets of sound objects per sound object category and three different categories of sound objects resulting in 36 number of trials of the attend sound object task in each run (3 attended objects × 4 sound scene types × 3 sound object categories). Similarly, as in the 3OA experiment, the 12 sound scene types were also presented during the visual control task but with different exemplars.

Note that trial composition and trial order was separately randomized for each participant.

### Experimental session

During fMRI, every participant started the session with the OA experiment. Half of the participants completed the 3OA experiment second and the 3OW experiment third. The other half completed the 3OW experiment second and the 3OA experiment third. For each experiment, four runs were completed, making 12 total experimental runs. Each run in the OA experiment included 36 trials and lasted ∼6 min. For the 3OA and 3OW, each run consisted of 48 trials lasting ∼8 min. In between runs, the participant was given a small break and the opportunity to communicate with the researchers. After the experimental runs, anatomical images were collected from participants.

### Pretrial

Before the fMRI session, participants practiced the experiments outside of the scanner. Each experiment was practiced for a total of two runs. Initially, each experiment was practiced with more lenient response times. Subsequently, a second set of practice runs was completed at a pace consistent with the actual paradigm. To mitigate potential learning effects, the practice runs used stimuli that were left out from the actual fMRI experiments.

### Equipment and stimulus delivery

Stimuli were presented with Presentation 24.0 software (Neurobehavioral Systems). The auditory stimuli were presented binaurally through earphones (Sensimetrics model S14; Sensimetrics). To minimize the head movement and to reduce the volume caused by the fMRI scanner, a piece of foam padding was placed over participants’ ears. Auditory volume was adjusted individually for each participant before the session at ca. 85 dB. Visual stimuli were projected onto a mirror attached to the head coil; no eye-tracking was performed. The participant answered the questions with an fMRI-compatible response pad with either the index, middle, or ring finger of their right hand which was placed on a foam padding.

### fMRI data acquisition

We collected functional imaging data with 3T MAGNETOM Skyra whole-body scanner (Siemens Healthineers) using a 32-channel head coil, with 2 coils removed to make the stimuli visually accessible. We used T2* echo planar imaging which collected 45 continuous oblique axial slices (TR, 1,050 ms; TE, 30 ms; flip angle, 75°; field of view, 21 cm; slice thickness, 2.7 mm; in-plane resolution, 2.7 mm × 2.7 mm × 2.7 mm). Each session consisted of 12 runs. For the first four runs (object-alone experiment), we collected ∼360 volumes per run, whereas for the last eight runs (3OA/3OW experiments), ∼570 volumes were collected per run. After the functional imaging, we collected a high-resolution T1 anatomical image (TE, 3.3 ms; TR, 2,530 ms; voxel matrix, 256 × 256; in-plane resolution, 1 mm × 1 mm × 1 mm) for coregistration. Each fMRI session lasted ∼100 min.

### Preprocessing of fMRI data

Preprocessing of the fMRI data was performed using FEAT (FMRI Expert Analysis Tool) version 6.00, part of FSL (FMRIB’s Software Library; http://www.fmrib.ox.ac.uk/fsl). Registration of fMRI volumes to the high-resolution structural image of the participant was carried out using FLIRT (Jenkinson and Smith, 2001; Jenkinson et al., 2002), and preprocessing included motion correction using MCFLIRT (Jenkinson et al., 2002), slice-timing correction, nonbrain removal with BET (Smith, 2002), and high-pass temporal filtering (with a cutoff of 100 Hz). For all further fMRI analyses except the spatial pattern analysis (see below, Spatial pattern analysis), the data were then projected to the FreeSurfer (Fischl, 2012) average surface space (fsaverage) using the FreeSurfer function mri_vol2surfs.

To generate confound regressors for the first-level analyses, we used *fMRIPrep* 20.2.5 (Esteban et al., 2018). Confounding time series for framewise displacement (FD) were calculated based on the preprocessed BOLD. FD was computed using two formulations following Power (absolute sum of relative motions; Power et al., 2014) and Jenkinson (relative root mean square displacement between affines; Jenkinson et al., 2002). FD was calculated for each functional run, both using their implementations in Nipype (following the definitions by Power et al., 2014). A global signal confound time series was extracted within the whole-brain masks. Additionally, a set of confounding regressors (the four first accounting for the majority of variance) were extracted to allow for component-based noise correction (aCompCor; Behzadi et al., 2007). Gridded (volumetric) resamplings were performed in a single interpolation step using antsApplyTransforms (ANTs), configured with Lanczos interpolation to minimize the smoothing effects of other kernels (Lanczos, 1964). Finally, six motion correction parameter time series were added as confound regressors.

### Experimental design and statistical analyses

#### Analysis of behavioral data and stimulus features

We used *JASP* (0.18.3, https://jasp-stats.org/download/) to analyze behavioral data from participants. All misses were interpreted as incorrect in the match-to-sample task and removed from the self-rating. The mean task performance (in both subjective ratings and match-to-sample) and standard error of mean were used to establish that the participants were performing the task as expected. To analyze participants’ performance during the attend speech object task across experiments, we performed a repeated-measures analyses of variance (ANOVAs) separately for the rating and match-to-sample tasks. The ANOVAs included two factors: Experiment (OA, 3OA, 3OW) and Sound object category (speech, animal, instrument). The performance during the visual control task was analyzed with repeated-measures ANOVAS with the factor Experiment (OA, 3OA, 3OW). We report *η_p_*^2^ and Greenhouse–Geisser adjusted dfs when sphericity was violated.

For each stimulus acoustic feature (see below, Stimulus acoustic features), we used *JASP* to analyze whether sound object categories differed in their acoustic feature (each sound object exemplar acted as a data point). Repeated-measures ANOVA was used with the factor Sound object category (speech, animal, instrument). We report *η_p_*^2^ and Greenhouse–Geisser adjusted dfs when sphericity was violated.

#### First- and second-level analysis of fMRI data

In the first-level analyses, a general linear model (GLM) was fit to the time series data of each voxel in each run, using FEAT (3 columns format). A separate GLM was run for the two sound scene experiments (3OA and 3OW). All GLMs included three regressors (one for each sound object category) for the timepoints when performing the attend sound object task. The attended sound object delineated the sound object category. For example, if the participants attended to the Husky sound object in a scene comprising Husky, Male1 and Trumpet, the timepoints were included in the attend sound object task animal regressor. We included nuisance regressors for all match-to-sample task timepoints. We also included one regressor for the visual control task in 3OA and three regressors (one for each sound object category) in the 3OW.

In 3OW, to estimate differences in stimulus-dependent processing of the respective sound scene types (speech, animal, instrument), we defined pairwise contrasts between the three different sound scenes presented during the visual control task, during which participants did not pay attention to the sounds. Thereafter, we defined interaction effects for ANOVAs with the factors Experiment (3OA, 3OW) and Task (attend sound object, visual control), separately for each sound object category. These interaction effects are conceptually the same as a pairwise comparison between ARMs in the 3OA experiment and the 3OW experiment. Finally, we wanted to estimate differences in ARMs between the different attended object category types (e.g., which regions show stronger ARMs for speech than animal sounds). In 3OA, we simply defined the pairwise contrasts between the three different categories of the attend sound object task (attend to speech, attend to animal, attend to instrument). However, in *3OW* both the stimuli and the focus of attention differed between the three different attend sound object tasks. Thus, to yield comparable results with those from 3OA, the same pairwise comparisons were performed after first contrasting each attend sound object task category with its respective visual control task.

All pairwise comparison contrast maps were averaged over the four runs.

#### Group-level univariate analysis of fMRI data

Group-level univariate statistics were based on a two-level procedure using a one-sample *t* test performed using the glm-fit function of the FreeSurfer software. Clusters were defined using permutation inference (a robust method for controlling false discoveries; Greve and Fischl, 2009) in FreeSurfer, with the initial cluster forming threshold *z* = 3.1, cluster probability *p* < 0.01 for the univariate analyses.

#### Spatial pattern analysis

We further tested whether selective attention in the 3OW and 3OA experiments elicits similar neural patterns as when the attended object was presented in isolation in the OA experiment. We ran a spherical searchlight analysis (radius 6 mm; with a mean 76, SD = 31, mode = 93 number of voxels) separately for each subject and each trial in 3OW/3OA. For each searchlight voxel, we regressed the spatial pattern in 3OW/3OA with the spatial patterns from OA for the corresponding stimuli. Thereafter we tested whether the OA pattern of a given stimulus better explained the pattern in 3OW/3OA when that stimulus was attended compared with when it was not attended.

Specifically, each voxel’s time series was first divided by its run-specific mean. Note that we did not normalize across trials or voxels to include as few additional steps as possible and because some studies advice against them (Garrido et al., 2007; Ritchie et al., 2021). Then, for a given voxel *V* and trial *T* in 3OW/3OA, the spatial pattern *P* related to that trial was taken by averaging volumes 3rd to 8th (from trial onset) and taking the spatial searchlight (6 mm radius) pattern around voxel *V*. The corresponding patterns for the isolated presentation of each of the three stimuli in trial *T* were taken from the OA experiment and used as regressors (3 regressors + constant) to gain separate estimates for how well the attended (Att) and distractor (Dist1, Dist2) stimuli explain the pattern *P*. This was repeated for each voxel, trial, and participant. We then averaged separately the Att, Dist1, and Dist2 beta values across trials within each category (speech, instrument, animal), experiment (3OW and 3OA), and participant, resulting in whole-brain spatial pattern maps for each category when they were attended as well as when they were distractors. Note that Dist1 and Dist2 are kept separate, as in 3OA they separate which category was attended (e.g., Dist1 β-values for speech depict speech as a distractor when animals are attended, and Dist2 β-values when instruments are attended). Thereafter, we subtracted from the mean category-specific Att*-*values the mean of that same sound category’s values when they were distractors (Dist1s and Dist2s of that category; asDist1 and asDist2). Finally, to test whether category-level patterns (mean of the OA patterns of, e.g., all instruments) can explain the results, we ran the same analyses with these patterns added as regressors, thus having six regressors (three exemplars, three categories) in 3OA, and four regressors (three exemplars, one category) in 3OW.

Group-level conjunction maps were performed in four steps: First, each spatial pattern difference map (Att minus asDist) was projected to the FreeSurfer average (fsaverage) using the participants’ own FreeSurfer surface (surface smoothening: 5 mm^2^ full-width at half-maximum smoothening). Thereafter, for each spatial pattern difference map, we performed a one-sample *t* test using the glm-fit function of the FreeSurfer software and defined clusters using permutation inference in FreeSurfer, with the initial cluster forming threshold *z* = 2.3, cluster probability *p* < 0.05. Finally, we calculated the overlapping clusters for two spatial pattern difference maps (Att vs asDist1 and Att vs asDist2) for each of the three sound categories.

#### Bayesian region of interest analysis

We used the significant clusters (initial cluster threshold, *z* = 3.1 permutated cluster significance, *p* < 0.01) to define our ROIs for Bayesian analyses. We calculated the mean BOLD signal for each condition/participant/ROI in the dataset that did not yield the significant cluster. Bayesian pairwise comparisons and ANOVAs were defined in *JASP*, and we report bf_10,incl_ for the effects of interest.

### Stimulus acoustic features

Acoustic features were calculated with MIRtoolbox (v 1.8.1.) running in Matlab (2023b). Six features were calculated: pitch, centroid, harmonic-to-noise ratio (HNR), amplitude modulation standard deviation (AMSD), frequency modulation standard deviation (FCSD), and entropy. For all features, a single value was derived for each clip. Pitch was calculated with the default autocorrelation method of the mirpitch-function, selecting the strongest periodic component (limited between 75 and 10,000 Hz). Centroid (mircentroid) is the spectral centroid, the mean of the spectrum of the audio. HNR is calculated as the ratio of the strongest periodic component (highest autocorrelation) to the aperiodic component (Boersma and Weenink, 2001; Leaver and Rauschecker, 2010). AMSD represents the variation in the sound amplitude across time and was calculated as the standard deviation of the absolute value of the sound signal after resampling to 60 Hz. FCSD is the spectral variation in the stimulus, calculated as the standard deviation of power across frequency spectrum. Entropy is the relative Shannon entropy of the spectrum of the stimulus (mirentropy). Because of skewedness, the pitch, frequency centroid, and AMSD were logarithmically transformed.

#### Estimation of effects of acoustic features on attention-related modulations

For the acoustic features (see Stimulus Acoustic features), where the three sound object categories differed, we ran fMRI regression analyses. Unlike prior studies which have included all acoustic features and categorical variables in the same GLM, we chose to run each analysis separately. This was chosen because of strong multicollinearity and negative correlations between the features, which would have yielded possibly uninterpretable results. This was conducted so that for each attended object, we extracted its demeaned acoustic feature value. Thereafter we ran fMRI regression analyses with one regressor of interest, where each value was the demeaned acoustic feature for the attended sound object in the scene, separately for each acoustic feature. Each GLM included the same confounds as our general univariate analyses (see above, First- and second-level analysis of fMRI data).

## Results

The three experiments were designed to yield complementary information about differences in ARMs between the three types of sound object categories. The 3OA experiment was designed to reveal how selective attention modulates processing when attending to objects in scenes that comprise different categories of objects. In contrast, 3OW was designed to reveal how selective attention modulates processing in scenes that comprise sound objects from the same object category. Furthermore, because in 3OW each scene composed of sound objects from only one category, we used data from this experiment to estimate how stimulus-dependent processing differed between the three categories of sound (see below). The OA experiment was used as a point of reference for behavioral performance (see below, Behavioral performance), as well as in our spatial pattern analyses, to yield spatial activation patterns for single objects (see below, Attention-driven modulation of spatial activation patterns).

### Behavioral performance

**Figure 2.**
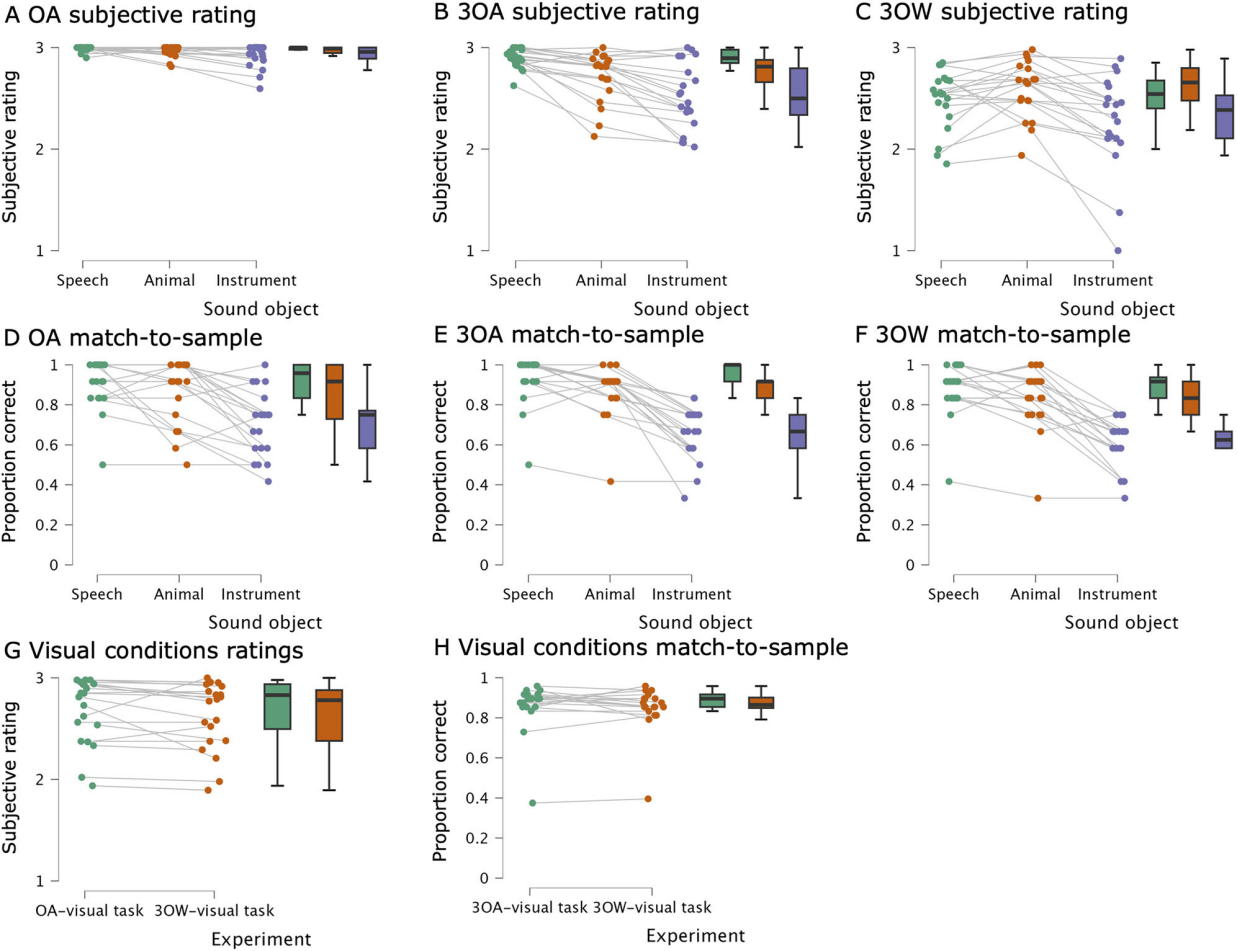
Behavioral results from the three experiments (OA, 3OA, 3OW). Participants gave both subjective ratings about their ability to attend to the stimuli and performed a match-to-sample task. In the rating task participants were instructed to rate how well they could focus on the attended stimuli with a scale 1–3 (1, <33% of the time; 2, 33–66% of the time; and 3, >66% of the time). In the match-to-sample task, participants were instructed to indicate whether a short excerpt matched the attended object or not. Each subplot first depicts the participants’ behavior across conditions (each line corresponds to a separate participant) and thereafter box plots for each condition (median, ±25 percentiles and ±47.5 percentiles). ***A–C***, Subjective ratings for each sound object type separately for the three experiments. ***D–F***, Performance in the match-to-sample task for each sound object type separately for the three experiments. ***G***, ***H***, Subjective ratings and performance in the match-to-sample task during the visual control conditions separately for the 3OA and 3OW experiment, respectively.

To study task performance in the three experiments (OA, 3OA, 3OW), we used subjective ratings and match-to-sample tasks (see Materials and Methods, Trial structure and Fig. 1). Our aim in the behavioral analyses was to evaluate whether participants successfully attended to the sound objects and whether attending to any of the object categories stood out in difficulty. To evaluate whether subjective ratings depended on the experiment and attended sound object category, we ran a repeated-measures ANOVAs with factors Experiment (OA, 3OA, 3OW) and Sound object (speech, animal, instrument; Fig. 2*A–C*). There was a significant main effect of Experiment (*F*_(2,38)_ = 46.4, *p* = 6.32 × 10^−11^, *η_p_*^2^ = 0.71), as participants found attending to the designated object easiest in OA (*μ* = 2.95, SEM = 0.046), thereafter in 3OA (*μ* = 2.71, SEM = 0.046), and most difficult in 3OW (*μ* = 2.45, SEM = 0.046; see Suppl. Text 1 for post hoc contrasts). There was a significant main effect of Sound object (*F*_(1.5,38)_ = 20.6, *p* = 5.76 × 10^−6^, *η_p_*^2^ = 0.52), as participants rated speech and animal sound objects (*μ* = 2.78, SEM = 0.041; *μ* = 2.75, SEM = 0.041) easier to attend to than the instrument sound objects (*μ* = 2.57, SEM = 0.041; see Suppl. Text 1 for post hoc contrasts contrasts). There was also a significant Experiment × Sound object interaction (*F*_(2.4,38)_ = 8.68, *p* = 4.04 × 10^−4^, *η_p_*^2^ = 0.314), because the pattern of differences in perceived difficulty between the three sound object categories varied across experiments: In 3OA, the differences were larger than in OA for speech versus animal and speech versus instruments, but not for animal versus instruments. In 3OW, the differences were larger than in OA only for animal versus instruments (see Suppl. Text 1 for post hoc contrasts). Thus, the different sound object categories were not rated consistently across experiments.

To evaluate whether participants performance in the match-to-sample tasks depended on the experiment and the sound object category, we ran a repeated-measures ANOVAs with the same factors as for the ratings (Fig. 2*D–F*). There was a small but significant main effect of Experiment (*F*_(2,38)_ = 4.3, *p* = 0.021, *η_p_*^2^ = 0.19), as participants performed worse in 3OW (*μ* = 0.76, SEM = 0.025), than OA and 3OA (*μ* = 0.83, SEM = 0.025; *μ* = 0.82, SEM = 0.025; see Suppl. Text 1 for post hoc contrasts). There was also a significant main effect of Sound object (*F*_(1.4,38)_ = 95.3, *p* = 1.26 × 10^−13^, *η_p_*^2^ = 0.83), as participants performed better with speech and animal sound objects (*μ* = 0.82, SEM = 0.025; *μ* = 0.81, SEM = 0.025) than the instrument sound objects (*μ* = 0.75, SEM = 0.025; see Suppl. Text 1 for post hoc contrasts). There was no significant Experiment × Sound object interaction (*F*_(3.2,38)_ = 1.36, *p* = 0.269, *η_p_*^2^ = 0.067; bf_10_ = 0.76 indicating weak evidence for no interaction being present). That is, differences in performance across object categories (Speech > Animal > Instrument) were comparable across experiments. This suggests that the difficulty differences in the match-to-sample task between the sound objects were not due to differential attentional demands for any of the sound categories in the scene experiments (3OA, 3OW), but rather because animal and instrument sounds were more difficult to recall during the match-to-sample period that the speech objects.

To evaluate whether the behavior in the visual control task depended on experiment, we ran for both subjective ratings and match-to-sample tasks a repeated-measures ANOVAs with the experiment (3OA, 3OW) as a factor. These ANOVAs revealed that there were no differences in subjective ratings (*F*_(1,19)_ = 2.32, *p* = 0.14, *η_p_*^2^ = 0.11), nor match-to-sample performance depending on the Experiment (*F*_(1,19)_ = 0.28, *p* = 0.61, *η_p_*^2^ = 0.015; Fig. 2*F*,*G*).

### Stimulus-dependent processing of sound scenes in AC

**Figure 3.**
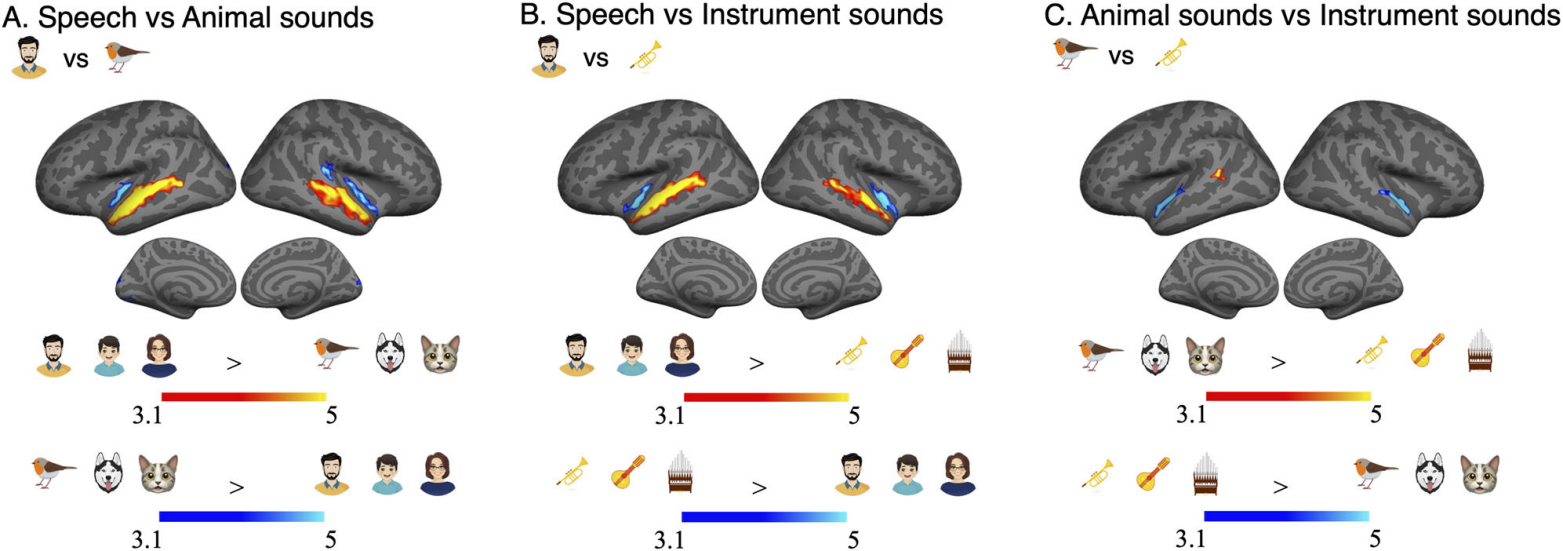
Stimulus-dependent processing of the three sound object category scenes (speech, animal, instrument) was estimated using pairwise comparisons between the three different auditory scenes presented during the visual control task in *3OW* (participants focused on the visual stimuli and ignored the auditory scenes). Initial cluster threshold was *z* = 3.1; permutated cluster significance *p* < 0.01. ***A***, Differences in stimulus-dependent processing between speech (red and yellow) and animal (blue and turquoise) sound objects. ***B***, Differences in stimulus-dependent processing between speech (red and yellow) and instrument (blue and turquoise) sound objects. ***C***, Differences in stimulus-dependent processing between animal (red and yellow) and instrument (blue and turquoise) sound objects.

First, we wanted to determine whether there were significant differences in the processing of stimulus features between the three categories of sound objects (speech, animal, instrument) in AC, when the objects were not the focus of attention. To examine such stimulus-dependent processing, we conducted pairwise comparisons between the three different auditory scene types (scenes comprising only speech, animal or instrument sounds) presented during the visual control task in 3OW, where participants focused on the visual stimuli and ignored the auditory scenes. In these comparisons all significant differences between the sound scenes were confined to the AC [for the definition and parcellation used for AC subfields, see Fig. S1 and for the definition and parcellation used for AC subfields, see Fig. S1 and Glasser et al. (2016)]. Speech sound scenes were associated with significant stimulus-dependent processing in all STG/S subfields, as well as lateral subfields A4 and A5, when comparing to the other two sound object categories (Fig. 3*A,B*, yellow and red). In contrast, animal and instrument sound scenes activated medial AC subfields MBelt, 52, and PI more strongly than speech sound scenes (Fig. 3*A,B*, blue and turquoise), while instrument sound scenes activated STGa, Ta2, and MBelt more strongly than animal sound scenes (Fig. 3*C*, blue and turquoise).

### Differences in attentional modulation of sound objects

Next, we delineated sound category-selective ARM clusters separately for the two experiments (3OA, 3OW) using pairwise comparisons between the three respective object categories (speech, animal, instrument). In 3OA, ARMs can be estimated simply by performing pairwise comparisons between the respective conditions, since the auditory scenes always comprised three objects from three different object categories and only the focus of attention varied between the conditions. In contrast, in 3OW this is not the case, because the auditory scenes always comprised one category of objects, and thus, direct comparisons might be dominated by stimulus-dependent activity. Therefore, to yield comparable results with those from 3OA, in 3OW we extracted ARMs for each sound object category by first contrasting each attend to sound object task condition with its corresponding visual control task condition. Because each auditory scene (e.g., male speaker, child, female speaker) was presented both during the attend sound object task and visual control task, any stimulus-dependent processing should be factored out in these contrasts. Thereafter pairwise comparisons were performed similarly as for 3OA.

**Figure 4.**
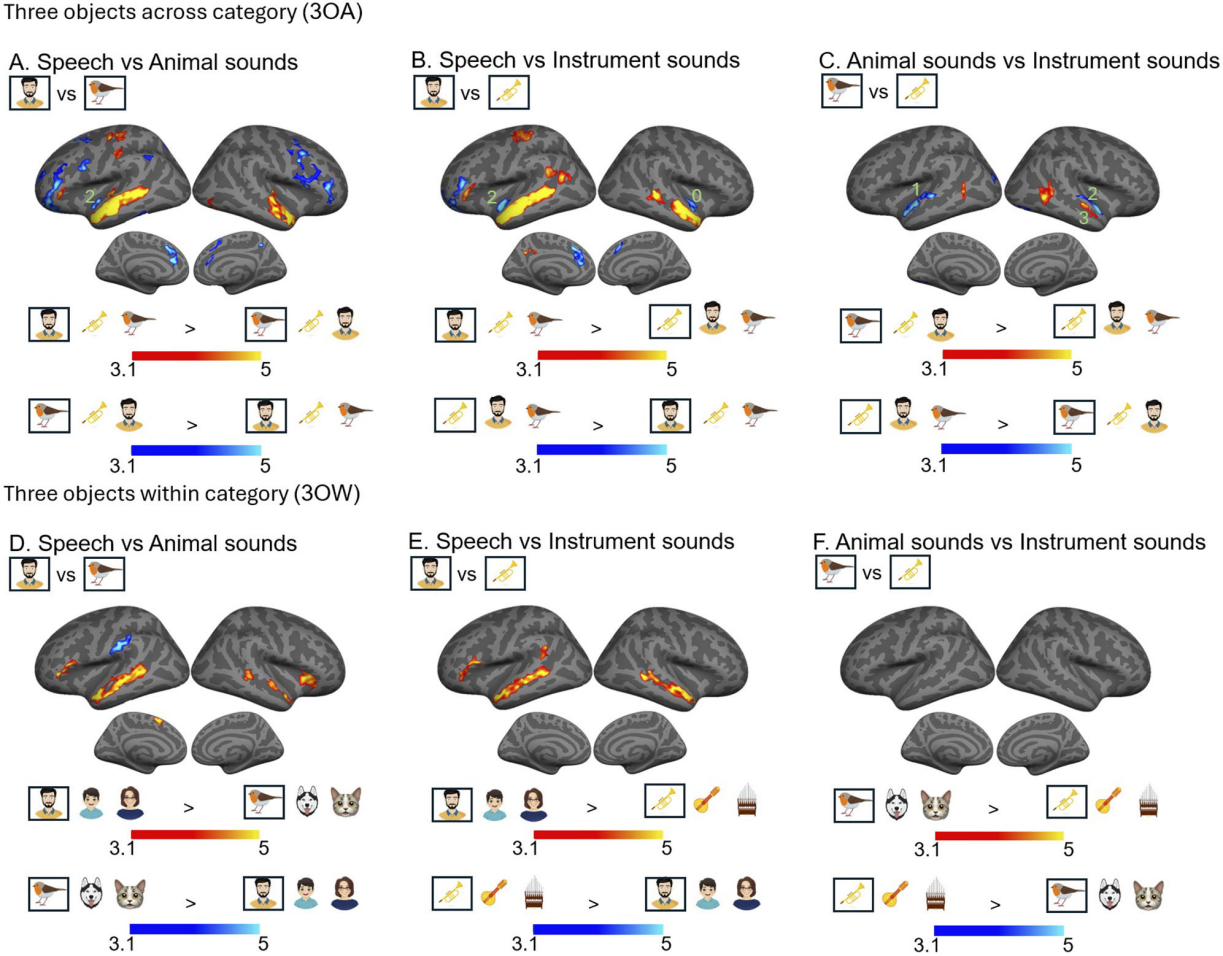
Pairwise comparisons reveal differences in attention-related activations between speech, animal, and instrument sound objects. Initial cluster threshold *z* = 3.1, permutated cluster significance *p* < 0.01, FWER. ***A***, ***D***, Differences in ARMs between speech and animal sound objects in 3OA and 3OW, respectively. ***B***, ***E***, Differences in ARMs between speech and instrument sound objects. ***C***, ***F***, Differences in ARMs between animal and instrument sound objects. Note that generally differences between sound object ARMs are observed in overlapping AC subfields as stimulus-dependent processing (Fig. 3). As some clusters in the AC were only observed in 3OA, we ran a Bayesian pairwise comparisons for 3OW data extracted from the cluster to test whether there was evidence for the same effect albeit subthreshold in 3OW. In these Bayesian pairwise comparisons, we denote Bayes factor (bf_10_), 0, bf < 1, negative evidence; 1, 1 < bf < 3, weak evidence; 2, 3 < bf < 20, Positive evidence; 3, 20 < bf < 150, Strong positive evidence. Thus, the number 2 in ***A*** denotes that there is positive evidence that animal sound object-related ARMs in this region are also present in the 3OW dataset (***D***).

Pairwise comparisons between the three object category ARMs separately for the two experiments (3OA, 3OW) are displayed in Figure 4 (speech vs animal sound objects, *A*,*D*; speech vs instrument sound objects, *B*,*E*; animal vs instrument sound objects, *C*,*F*). As can be observed by comparing the results displayed in Figure 4 with those in Figure 3, differences in ARMs between the three object categories were observed in similar AC subfields as differences in stimulus-dependent processing. However, some differences between the sound object ARMs in AC were observed in subfields where no stimulus-dependent processing differences were observed: stronger ARMs were observed for human sound objects than animal sound objects in A1 (Fig. 4*A*, red and yellow), and a cluster in the STSda showed stronger ARMs for animal than instrument sound objects (Fig. 4*C*, red and yellow). Outside the AC, significant differences between sound object ARMs were observed in inferior frontal and sensorimotor regions for speech objects (Fig. 4*A*–*C*, red and yellow) and various prefrontal, inferior parietal, medial frontal, and medial posterior subfields for the animal and instrument sound objects (Fig. 4*A*–*C*, blue and turquoise).

As was expected (see above), many of the smaller clusters observed in the 3OA experiment did not survive cluster correction in the 3OW experiment. Therefore, we extracted the significant AC clusters (that were not observed in the 3OW experiment) of the 3OA experiment and ran an ROI-based Bayesian analysis (see Materials and Methods, Bayesian region of interest analysis) for the 3OW data to test whether the ARM differed in these clusters between the experiments. As can be seen in Figure 4, there was generally positive–strong positive evidence (3 < bf_10_ < 20; 20 < bf_10_ < 150) that similar effects were present in the 3OW experiment (Fig. 4).

Note that the observed differences in the sound object category ARMs for 3OA and 3OW are unlikely to stem from performance disparities between those categories. First, the match-to-sample task revealed no significant Experiment × Sound object interaction. Second, participants’ subjective ratings of the object categories varied inconsistently across experiments, while the ARM differences were similar across experiments (for details, see above, Behavioral performance).

### Differences between attention-related modulations in 3OA and 3OW

**Figure 5.**
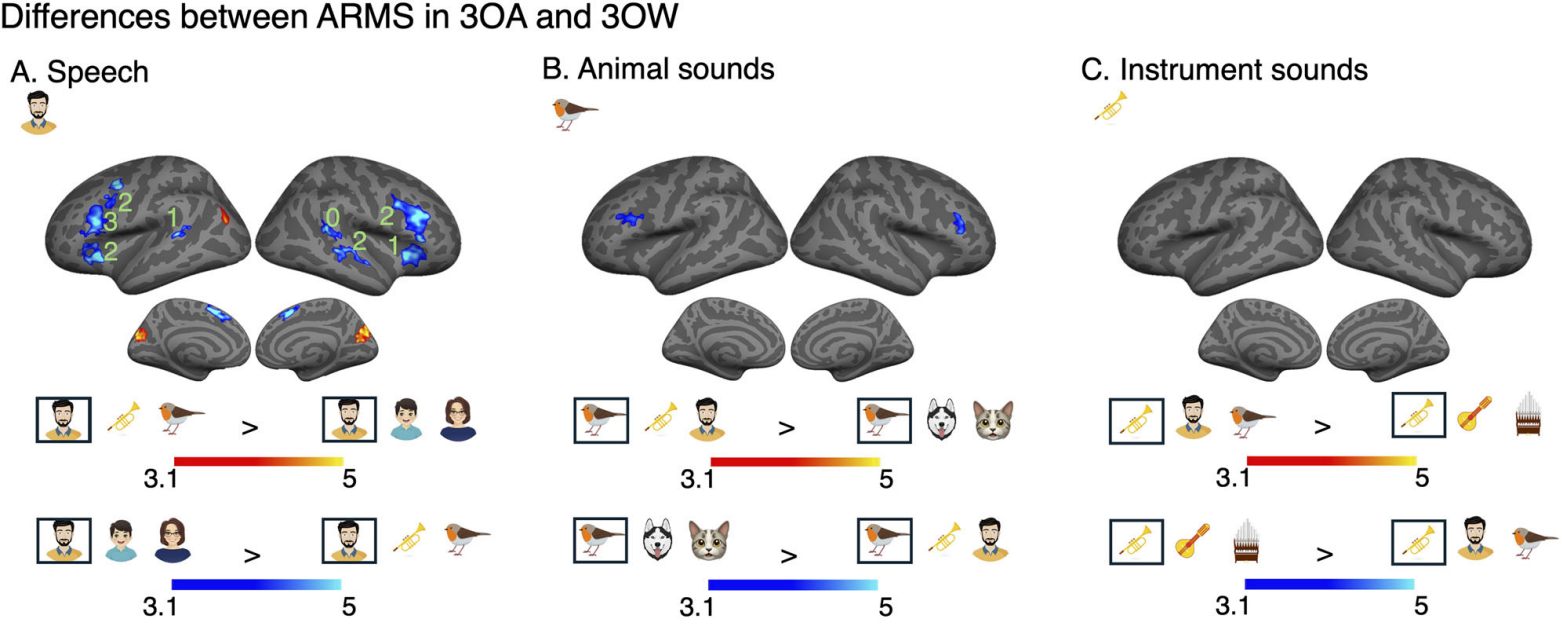
Repeated-measures ANOVAs reveal differences between ARMs in the two experiments (3OA > 3OW, red and yellow; 3OW > 3OA, blue and turquoise), performed separately for attended speech, animal, or instrument sound objects. Note that from these ANOVAs we only present the interaction effects, as they are conceptually the same as pairwise comparisons of the estimated ARMs in the two experiments (Fig. 4). Initial cluster threshold *z* = 3.1, permutated cluster significance *p* < 0.01. ***A***, Significant differences in ARMs for speech sound objects (red and yellow). ***B***, Significant differences in ARMs for animal sound objects. ***C***, Significant differences in ARMs for instrument sound objects. As some clusters were observed for only one of the three attended sound objects, we ran Bayesian repeated-measures ANOVAs [Experiment (3OA, 3OW) and Sound object (object1, object2)], for data extracted from the cluster to test whether there was evidence for the same effect, albeit subthreshold. In these Bayesian analyses, the same BF notation is used as in Figure 4. Thus, the number 3 in ***A*** denotes that there is strong positive evidence that ARMs are stronger in the 3OW than the 3OA experiment also for animal and instrument sound objects (***B***, ***C***).

We also wanted to determine whether ARMs differed when participants selectively attended to a sound object in auditory scenes comprising objects from the three different object categories (3OA) compared with when all objects were from the same object category (3OW). To this end, we inspected interaction effects from three separate (speech, animal, instrument) repeated-measures ANOVAs with factors Experiment (3OA, 3OW) and Task (attend sound object task, visual control task). This interaction effect is conceptually the same as a pairwise comparison between ARMs in the 3OA experiment and the 3OW experiment. As can be seen in Figure 5, ARMs were bilaterally stronger for 3OW than 3OA in the inferior frontal cortex for both speech and animal sound objects (Fig. 5*A*,*B*), as well as in anterior insula, and STG/STS regions for speech sound objects (Fig. 5*A*). In order not to make the wrong conclusion that the latter clusters were exclusive to speech, we ran Bayesian ANOVAs to evaluate whether there was evidence for similar ARM differences for the two categories that did not yield significant clusters. Thus, we extracted the significant clusters and performed Bayesian ROI analyses for the two sound object categories that did not yield significant clusters (see Bayesian region of interest analysis). In general, the clusters found for speech yielded positive–strong positive evidence (3 < bf_10_ < 20; 20 < bf_10_ < 150) for other sound objects (Fig. 5).

### Attention-driven modulation of spatial activation patterns

**Figure 6.**
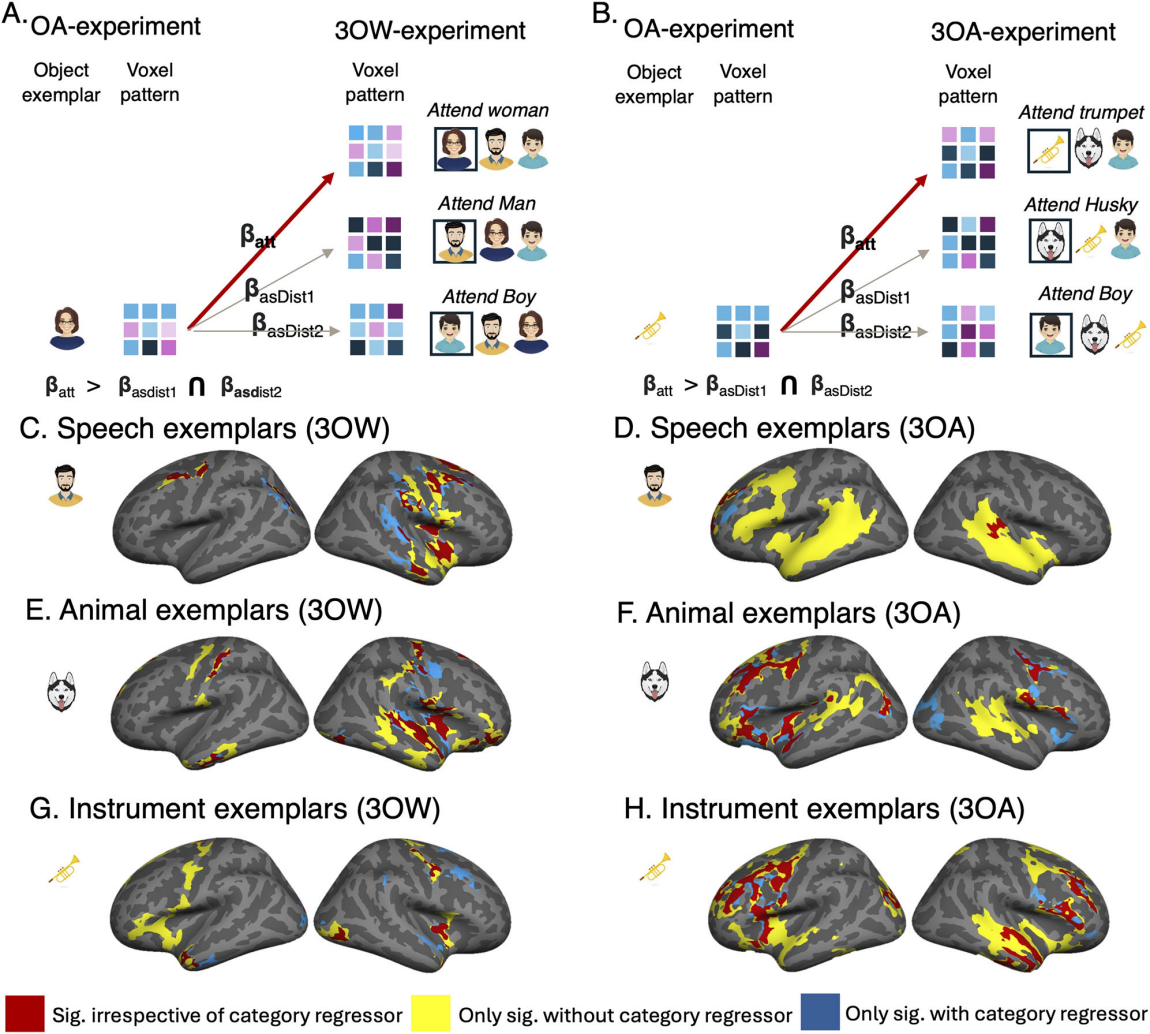
We ran searchlight analyses to evaluate whether activation patterns in the scene experiments (3OA and 3OW) could be predicted using the activation patterns elicited by the constituent object exemplars when presented alone in the OA experiment. Thereafter, we evaluated using combination analyses whether the scene related activation pattern was dominated by the attended object exemplar. ***A***, We used the voxel pattern of each of the three object exemplars present in the scene (in this illustration man, distractor1; boy, distractor2; woman, attended) from OA and used these three voxel patterns to predict the voxel pattern of the full scene. To evaluate whether the correlates were explained by category-level activation patterns, we also performed the same regression analyses controlling for category-level activation patterns (i.e., we added a regressor for the voxel pattern across all respective speech, animal or instrument exemplars). These procedures were performed for each scene, whereafter we calculated average regression maps for speech, animal, or instrument objects when they were attended (*β*_att_) and scenes where the objects were distractors (*β*_asdist1_, *β*_asdist2_). Hereafter, we calculated two pairwise comparisons for each object category separately (*β*_att_ > *β*_asdist1_) and (*β*_att_ > *β*_asdist2_) and show voxels where both comparisons yielded significant clusters (initial cluster threshold *z* = 2.3, permutated cluster significance *p* < 0.05). The results of such combination analyses are displayed separately for each object type in ***C***, ***E***, and ***G***. Red denotes clusters where the attended object dominated activation patterns irrespective of whether category-level activation patterns were controlled for or not; yellow where the analysis was not significant when controlling for category-level activation patterns; blue where only the analysis including category-level activation patterns were significant. ***B***, For 3OA the logic was the same as for 3OW.

**Figure 7.**
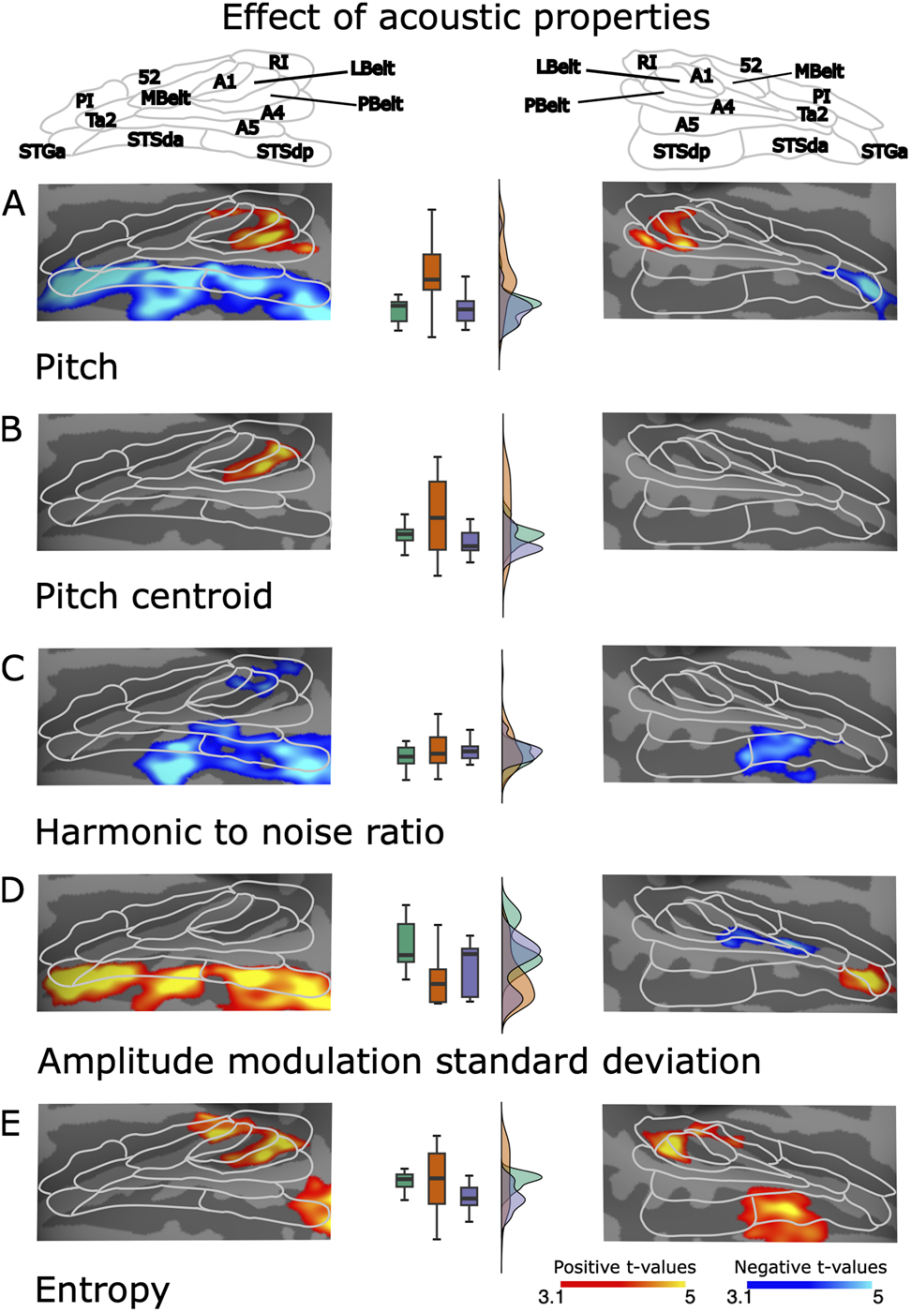
To examine the extent to which our category-selective ARMs were influenced by acoustic features of the attended sound objects, we performed separate regression analyses for each acoustic feature, derived for the attended object of the sound object scenes. At the top of the figure, we show AC subfields as defined in the HCP-parcellation (Glasser et al., 2016 CC BY 4.0; https://figshare.com/articles/dataset/HCP-MMP1_0_projected_on_fsaverage/3498446). In each subplot (***A–E***), red and yellow denote significant positive correlations, and blue and turquoise denote significant negative correlations. In the middle of the subplots, we show box and distribution plots of the respective auditory sound object category (speech, animal, instrument) acoustic feature values. Initial cluster threshold *z* = 3.1, permutated cluster significance *p* < 0.01.

To evaluate whether the focus of attention not only affected mean BOLD signal but also spatial activation patterns, we determined whether the voxel patterns present in scenes comprising three separate objects (in 3OW and 3OA) were dominated by the voxel pattern of the attended object, at the cost of the distractor objects. Thus, we derived voxel patterns (6-mm-radius searchlight) for each sound object exemplar from OA experiment (where each object was presented in isolation) and used these voxel patterns as regressors to predict voxel patterns of scenes with the same sound object exemplars in the 3OW and 3OA experiment (for details on this analysis, see Materials and Methods, Spatial pattern analysis). For example, in the 3OW experiment for a scene where exemplar no. 1 of the woman speech object was attended and exemplar no. 8 of the boy speech object and exemplar no. 4 of the male speech object were distractors, we derived the voxel pattern for each of these three object exemplars from the OA experiment and used these voxel patterns as regressors for the voxel pattern of the full scene. To evaluate whether the attended object exemplars voxel pattern dominated the voxel pattern of the whole scene, we performed combination analyses (see Materials and Methods, Spatial pattern analysis and Fig. 6 for details). Additionally, we ran the same analyses while controlling for general object category activation patterns (i.e., voxel pattern across all exemplars of the category; see Materials and Methods, Spatial pattern analysis and Fig. 6 for details). The same analysis was also performed for the Glasser AC ROIs (depicted in Fig. 7), which is reported in Text S2, Table S3, and Figure S2.

As can be seen in Figure 6*C–H*, the way attention-modulated spatial activation patterns depended strongly on both experiment (3OW and 3OW) and attended object (speech, instrument, animal) as well as on whether category-level patterns were controlled or not. For speech, in 3OA the attended exemplar dominated voxel patterns in extensive bilateral AC fields, temporal cortex, as well as left inferior–lateral frontal regions (Fig. 6*D*, yellow and red). However, when controlling for the average speech-related activation pattern, only the right posterior AC and left lateral frontal regions remained significant (Fig. 6*D*, blue and red; Fig. S2*B*). In contrast, in 3OW the attended speech exemplar dominated voxel patterns in right AC fields (TA2, STGa, STSda, PI), as well as right middle temporal gyrus, right insular fields, right temporal pole, and bilateral inferior parietal–sensorimotor regions (Fig. 6*C*, yellow and red). When controlling for the average speech-related activation pattern, the exemplar-related pattern in the right AC was skewed posteriorly to subfields PBelt, A4, and A5 (Fig. 6*C*, blue and red).

For animals, in both experiments the attended object dominated activation patterns in similar anterior and posterior AC subfields as observed for speech in 3OW (Fig. 6*E*, red and yellow; Fig. S2*C,D*). Furthermore, in 3OA the right hemisphere cluster extended beyond the AC to STG/STS regions. Outside the AC attended animal exemplars dominated voxel patterns in middle temporal gyrus (extending to the temporal pole in 3OW) as well as in bilateral inferior parietal–sensorimotor regions and the right insula, extending to inferior frontal cortex (Fig. 6*E*,*F*, red and yellow). Note that as for speech, many of the AC clusters were not significant when controlling for average animal spatial pattern in 3OA (see also Fig. S2*D*).

For instruments, in 3OA the attended instrument exemplar dominated activation patterns bilaterally in AC fields Ta2 and PI as well as parts of A4, A5, MBelt, and right hemisphere STG/STS regions (Fig. 6*H*, red and yellow; Fig. S2*F*). Outside the AC the attended instrument exemplars dominated spatial patterns in bilateral inferior frontal, bilateral insular, left lateral frontal, and right middle temporal gyrus extending to the temporal pole (Fig. 6*H*, red and yellow). When controlling for average instrument spatial patterns, only a cluster in the right posterior superior temporal gyrus remained significant within the AC. In 3OW, the attended instrument exemplar dominated similar bilateral AC spatial activation patterns as in 3OA (albeit right AC subfields only emerged when controlling for average instrument activation patterns; Fig. 6*G*, red and yellow; Fig. S2*E*). Outside the AC attended instrument exemplars dominated spatial activation patterns in bilateral insula, lateral visual cortex, left temporal pole, left inferior frontal, and sensorimotor regions.

### Effects of acoustic features on attention-related modulations

All sound object categories were matched by duration and perceptual loudness. In addition, speech and instrument sound object categories were rendered highly similar on other acoustic features, because the instrument sound object exemplars were generated from speech sound object exemplars of the same speakers as those that constituted our speech sound objects (see Materials and Methods, Stimuli). However, the animal sound object exemplars were gathered from heterogeneous sources (see Materials and Methods, Stimuli) making it impossible to fully match acoustic features of the animal sound object category with the speech and instrument categories. Therefore, as in previous studies (Leaver and Rauschecker, 2010), we assessed whether the categories differed on several acoustic features. Thereafter, for the features the sounds differed on, we examined the extent to which our category-selective ARMs were influenced by these features.

We used ANOVAs (Welsh) with the factor Sound object category (speech, animal, instrument) to test whether the object categories differed on any of the acoustic features (see Materials and Methods, Stimulus acoustic features). There were significant main effects of Sound object category for all studied acoustic features except FCSD (*F*_(2,85.7)_ = 0.25, *p* = 0.77, *η*^2^ = 0.004; Fig. 7, middle column): pitch (*F*_(2,85.3)_ = 21.2, *p* = 3.36 × 10^−8^, *η*^2^ = 0.28), frequency centroid (*F*_(2,87)_ = 16.8, *p* = 6.77 × 10^−7^, *η*^2^ = 0.2), HNR (*F*_(2,89)_ = 5.67, *p* = 0.005, *η*^2^ = 0.05), AMSD (*F*_(2,93.8)_ = 22.89, *p* = 8.02 × 10^−9^, *η*^2^ = 0.240), and entropy (*F*_(2,81.7)_ = 29.9, *p* = 1.80 × 10^−10^, *η*^2^ = 0.190).

As the sound object categories differed on five of the six acoustic features studied, we examined the extent to which our category-selective ARMs (Fig. 7) were influenced by the fact that the categories differed in their acoustic features. For this purpose, we ran a separate regression analysis (see Effects of acoustic features on attention-related modulations) for each of the five acoustic features on 3OA fMRI data, which was chosen as ARMs were more strongly category specific in this experiment (Fig. 4). Note that the value for each acoustic feature was derived for the attended object of the sound object scenes (see Effects of acoustic features on attention-related modulations). By comparing the results of these analyses (Fig. 7) with the category-selective ARMs presented in Figure 4, it can be discerned that some of the clusters, such as negative correlations for pitch and HNR (Fig. 7*A*,*C*, blue and turquoise), as well as positive correlations for AMSD (Fig. 7*D*, red and yellow), arose in the same regions where speech sound objects differed from the two other object categories (Fig. 4*A*,*B*,*D*,*E*). Thus, one could argue that the human-selective clusters displayed in Figure 4 arose because speech sounds differed on these acoustic features. However, because the same clusters arose for all the acoustic features, and the categorical effect (speech > animal, instrument; Fig. 4) was much stronger than any acoustic feature effect, we argue for the reverse. In contrast, the regression analyses for acoustic features also revealed correlations in AC subfields that were not observed for any ARM contrast between sound object categories. That is, pitch, pitch centroid, and entropy correlated positively with ARMs bilaterally in LBelt and PBelt (Fig. 7*A*,*B*,*E*, red and yellow); HNR correlated negatively with ARMs in RI, LBelt, and A1 (Fig. 7*C*, blue and turquoise); AMSD correlated negatively with ARMs in A4 (Fig. 7*D*, blue and turquoise); entropy correlated positively bilaterally in posterior parts of MBelt (Fig. 7*E*, red and yellow). Taken together, these results indicated that the categorical ARM effects were unlikely caused by differences in acoustic features. For further discussion regarding these effects, see Text S3.

## Discussion

We examined stimulus-dependent and attention-modulated AC representations for sound categories in scenes with overlapping objects. Consistent with previous work (Leaver and Rauschecker, 2010; Norman-Haignere et al., 2015), the sound categories activated distinct AC subfields regardless of attention. Specifically, speech activated higher-level lateral AC subfields, whereas animal and instrument sounds engaged higher-level medial subfields. Notably, attention enhanced activity in AC regions that also displayed stimulus-dependent responses for the attended category. These results support models where attention increases the gain of neurons encoding the attended sound’s features (Kerlin et al., 2010; Zion-Golumbic et al., 2013). Yet, our spatial pattern analysis suggests that gain modulation is just one mechanism by which attention transforms AC representations: In scenes with objects from different categories, the attended object dominated activation patterns in regions processing high-level, category-specific features. Conversely, in scenes where all objects were from the same category, attentional dominance shifted to AC fields processing low-level acoustic features. Thus, attention appears to prioritize features with maximal contrast, highlighting object-specific patterns in feature-similar scenes and category-level patterns in diverse scenes. Consequently, attention might modulate neuronal gain as well as auditory scene decomposition in AC (Griffiths and Warren, 2002, 2004; Angeloni and Geffen, 2018).

### Stimulus-dependent selectivity for speech, instrument, and animal auditory objects in AC subfields

We estimated stimulus-dependent processing by comparing activations from scenes containing only one category during the visual control task in 3OW (with attention directed to visual stimuli and sounds ignored). Lateral AC and STG/STS subfields were selective for speech, while medial AC subfields showed selectivity for instrument and animal sounds, corroborating models that speech processing diverges in higher-level AC (Hickok, 2010; Zhang et al., 2011; Friederici and Gierhan, 2013; Rolls et al., 2023). Our category-selective clusters align with fields previously identified as speech versus music selective (Norman-Haignere et al., 2015, 2022; Hausfeld et al., 2018). However, rather than conventional music, our instrument sounds were derived from human speech by extracting its pitch profile (see Materials and Methods, Stimuli). Consequently, our speech and instrument sounds shared comparable low-level acoustics and temporal structure (Fig. 7), unlike speech–music. Given limited data on instrument/animal-selective medial AC fields, their precise function remains unclear, though they may process higher-level nonspeech properties or integrate low-level feature information that differentiate such objects (Rauschecker and Scott, 2009). Furthermore, we observed that MBelt, LBelt, and TA2 showed stronger stimulus-dependent processing for instrument than animal sounds (Fig. 3*C*, blue and turquoise). A previous fMRI study (Norman-Haignere et al., 2015) using naturalistic sound clips and canonical response profiles found that sounds eliciting the strongest responses in these subfields featured pitch or spectral modulations indicative of harmonics. Thus, differences in harmonic structure between animal and instrument sounds may account for the greater selectivity for instrument sounds in these subfields (see also Hausfeld et al., 2018).

### Attentional selection of auditory objects depends on object type and the embedding scene

ARMs yielded object selectivity on par with stimulus-dependent processing. Specifically, medial AC subfields exhibited stronger ARMs for animal/instrument objects than for speech, whereas lateral AC subfields showed the converse. Previous research has consistently shown that selective attention to speech in multi-speaker scenes activates lateral AC subfields and STS regions (Alho et al., 2003, 2006; Leminen et al., 2020; Wikman et al., 2020, 2022; Ylinen et al., 2022). Our findings suggest that these effects are specific to speech and do not generalize to other objects.

Our findings that attention selectively modulated processing in the same AC subfields involved in stimulus-dependent processing of the attended sound object type support—but do not prove—models positing that attention alters AC gain and tuning parameters (Fritz et al., 2007; Kauramäki et al., 2007; Moerel et al., 2013). Consequently, we conducted a pattern analysis, in which we evaluated, whether the voxel patterns in scenes with three overlapping objects (3OW and 3OA) were primarily driven by the attended object, diminishing distractor representation. We reasoned that if attention merely enhances gain of neurons selective to the attended object, the pattern analysis would replicate our univariate ARM effects (Hausfeld et al., 2018; Puschmann et al., 2024). Moreover, if attention solely scales neuronal gain, its effects should be invariant to scene type. Yet, the influence of attention on activation patterns varied markedly with scene type (3OA vs 3OW), as follows.

For speech, the attended object dominated the activation pattern throughout the left hemisphere language network (Friederici and Gierhan, 2013; Fedorenko et al., 2024). However, this effect was limited to 3OA scenes. Conversely, in 3OW scenes—where both the attended object and distractors were speech—the analysis produced markedly different outcomes: In 3OW, attended speech predominated in the right hemisphere, especially in fields associated with low-level speech, spectral, and voice identity processing (Bonte et al., 2014; Norman-Haignere et al., 2015, 2022). Notably, after controlling for general speech category-level patterns, the broad language network correlates vanished in 3OA, whereas the effects in 3OW were more nuanced. These findings suggest that in 3OA, attention boosts general speech activation patterns, unlike 3OW. For animal and instrument sounds, effects were more comparable in the two experiments, albeit in 3OW effects were more strongly constrained to regions associated with spectral processing of sounds. Mirroring the effects seen for speech, the patterns for animal and instrument exemplars were more strongly affected by controlling for the sound object category in 3OA compared with 3OW. The differences were, however, less striking, possibly because animal and instrument exemplars might elicit more variable patterns than speech in AC.

The disparity in attentional dynamics between the experiments aligns with our hypothesis: In 3OW, attention targets lower-level features to segregate same-category objects, whereas in 3OA, category-level differences drive object separation. Moreover, these differences were not confined to our pattern analysis; univariate results also showed stronger ARMs in 3OW than in 3OA in regions sensitive to low-level spectral information (Fig. 5*A*). As performance was poorer in 3OW than 3OA, it could be argued that task difficulty affected the disparate AC patterns of the two experiments. However, general task difficulty has not consistently been associated with AC modulation (Rinne et al., 2009, 2011; Wikman et al., 2020). Therefore, we argue that behavioral and neural effects both relate to differences in specific task demands caused by the higher similarity among the three overlapping sounds in 3OW [see also Alho et al. (1992) and Woods et al. (1992) who showed that greater similarity between auditory targets and nontargets enhances attentional modulation of early evoked responses]. Thus, both behavioral and neural effects are consistent with the notion that neural networks differentiating sound categories may be distinct and only partially overlapping with those that distinguish individual exemplars (Giordano et al., 2013), with attention flexibly operating at different representational levels depending on the task at hand (Wikman et al., 2020, 2024).

The results from our 3OW pattern analysis differ markedly from the findings of Puschmann et al. (2024), where the activation pattern of two speech streams were used to predict the activation pattern of both speech streams heard simultaneously (one stream was attended, the other a distractor). The authors found that the attended input predicted activation patterns in bilateral AC and STS regions more strongly than the distracting input (i.e., activation patterns were modulated in regions where we found no correlates in 3OW). We argue these disparities might be explained by two factors: (1) Attention affects stream segregation in AC (Griffiths and Warren, 2002, 2004; Cusack et al., 2004; Cusack, 2005; Angeloni and Geffen, 2018) by “boosting” the neural processes that differentiate attended objects from distracting sounds, building the so-called attentional map in AC (Näätänen, 1990) representing properties that differentiate attended objects from distractors (Wikman et al., 2020, 2024). (2) When this map is stable, a second attention-driven phenomenon occurs: Attention starts gating higher-level information processing (semantic, linguistic, etc.) preferentially for attended speech and suppress such processing for distracting speech (Puschmann et al., 2024). Thus, as we used short speech segments (starting from the onset of the sound), attention was not yet gating exclusively the attended input for higher-level processing. In contrast, Puschman et al. used long segments (>11 min) and analyzed the last 10 min of the stream (when BOLD activity had plateaued); consequently the characteristics differentiating the two streams had probably already been established and such gating could occur.

The account presented in the previous paragraph might also explain some of the striking dissimilarities between speech attention effects on activation patterns in 3OA and 3OW. That is, when the distracting auditory input is nonspeech (3OA), the characteristics differentiating speech from the distractor sounds are immediately evident and the attentional map might be formed almost instantaneously and thus higher-level analysis of speech can be much more quickly gated by attention, explaining why correlates where observed across the language network. Thus, our study suggests that temporal aspects of attention is important to consider, i.e., selective attention may operate, context and task specifically, on several levels at different points in time.
